# Valsartan ameliorates ageing-induced aorta degeneration *via* angiotensin II type 1 receptor-mediated ERK activity

**DOI:** 10.1111/jcmm.12251

**Published:** 2014-02-18

**Authors:** HaiYan Shan, Siyang Zhang, Xuelian Li, Kai yu, Xin Zhao, Xinyue Chen, Bo Jin, XiaoJuan Bai

**Affiliations:** a Department of Gerontology, The First Affiliated Hospital of China Medical UniversityShenyang, Liaoning Province, China; b Center Laboratory Technology and Experimental Medicine, China Medical UniversityShenyang, Liaoning Province, China; c Department of Epidemiology, School of Public Health, China Medical UniversityShenyang, Liaoning Province, China

**Keywords:** vascular ageing, angiotensin II, valsartan, Bcl-2, Bax, mitogen-activated protein kinase

## Abstract

Angiotensin II (Ang II) plays important roles in ageing-related disorders through its type 1 receptor (AT_1_R). However, the role and underlying mechanisms of AT1R in ageing-related vascular degeneration are not well understood. In this study, 40 ageing rats were randomly divided into two groups: ageing group which received no treatment (ageing control), and valsartan group which took valsartan (selective AT1R blocker) daily for 6 months. 20 young rats were used as adult control. The aorta structure were analysed by histological staining and electron microscopy. Bcl-2/Bax expression in aorta was analysed by immunohistochemical staining, RT-PCR and Western blotting. The expressions of AT_1_R, AT_2_R and mitogen-activated protein kinases (MAPKs) were detected. Significant structural degeneration of aorta in the ageing rats was observed, and the degeneration was remarkably ameliorated by long-term administration of valsartan. With ageing, the expression of AT1R was elevated, the ratio of Bcl-2/Bax was decreased and meanwhile, an important subgroup of MAPKs, extracellular signal-regulated kinase (ERK) activity was elevated. However, these changes in ageing rats could be reversed to some extent by valsartan. *In vitro* experiments observed consistent results as *in vivo* study. Furthermore, ERK inhibitor could also acquire partial effects as valsartan without affecting AT1R expression. The results indicated that AT1R involved in the ageing-related degeneration of aorta and AT1R-mediated ERK activity was an important mechanism underlying the process.

## Introduction

Population ageing is now a worldwide problem. In the United States, there are 35 million people of more than 65 years old. It is estimated that the number will be doubled in the year 2030 [1]. In the process of ageing, many physiological functions will be impaired or degenerate, resulting in a number of detrimental consequences for human health [2,3]. Therefore, attenuating ageing-induced damage to human health is of great significance in improving the quality of human lives in the ageing society.

As is known, ageing will inevitably lead to the changes in the activity or responsiveness of hormonal systems, one of which is the angiotensin II (Ang II). Experiments in ageing animals demonstrated that selective blocking of Ang II type 1 receptor could significantly decrease the expression of senescence markers and thus retard the progression of ageing [4,5], indicating that Ang II played an important role in ageing-related pathologic processes and its type 1 receptor was an important mediator.

Cardiovascular disease is a typical one of diseases that are closely related to the ageing. It has been reported that ageing is the largest risk factor for cardiovascular disease [6,7]. Meanwhile, data have demonstrated that Ang II played an important role in vascular ageing as well as in the initiation and progression of atherosclerosis [8,9]. Therefore, in the past decades, independent groups have focused on Ang II as a mediator of vascular cell dysfunction in various cardiovascular disorders [10,11]. In addition, the mechanisms that involved in the Ang II-mediated vascular injury in several cardiovascular diseases were also partially revealed, *e.g*. Ang II causes arteriolar vasoconstriction, superoxide anion production and endothelin release through its type 1 receptor (AT1R), resulting in increased vascular resistance and promoting atherosclerosis [12]. However, in ageing-related vascular injury, the role and mechanisms of Ang II were yet unclear.

Valsartan is a selective Ang II receptor blocker (ARB). Over the past decades, experimental studies and clinical trials have demonstrated that valsartan seceratively blocked Ang II type 1 receptor (AT1R) and thus, it was widely used in treatment of Ang II-involved cardiovascular disorders, such as hypertension, endothelial dysfunction [12] and abdominal aortic aneurysm [13]. However, the potential effect of valsartan on normal ageing-induced vascular injury has not been investigated. Whether valsartan could attenuate the ageing-induced vascular injury and the underlying mechanisms still remains unknown.

In this study, we investigated the potential role and mechanisms of Ang II in the structural and functional degeneration occurring in the normal ageing. Then, we tested the hypothesis that long-term administration of valsartan would protect vessels from ageing-induced injury in a rat model of normal ageing and furthermore, we explored the underlying mechanism involving in the process.

## Material and methods

### Experimental animals

Twenty young (or adult, 3-month-old) and 40 aged (18-month-old) male Wistar rats were purchased from the Department of Laboratory Animals, China Medical University. Animals were maintained at controlled temperature of 21°C and in a 12-hour day/night cycle. All the experimental procedures were approved by the Institutional Animal Care and Use Committee of China Medical University.

Young or adult animals were used as control group. Aged animals were randomly divided into two groups: the ageing group and Valsartan group (*n* = 20 in each group). The control and the ageing animals had free access to water and standard rat chow. The valsartan group animals continually took valsartan (Novartis Pharma Stein AG; 30 mg/kg/day) in their drinking water for 6 months. The concentration of valsartan dissolved in the drinking water was determined based on the previously established rats drinking patterns.

### Isolation, culture and treatment of rat aorta endothelial cells

Isolation and characterization of aorta endothelial cells were performed according to the previous report with modifications [14]. Thirty minutes before the isolation of aorta, Wistar rats were intraabdominally injected with 6500 U Heparin Sodium. Then, animals were anaesthetized by injection of pentobarbital sodium. The thoracic aorta was identified and isolated. Connective tissues outside the aorta were removed. After washing in PBS, the minute arterial branch was removed and then vascular cells were isolated and cultured in DMEM (Gibco, Pascagoula, MS, USA) supplemented with 20% foetal bovine serum (Gibco).

For treatment of aorta endothelial cells, the culture medium was supplemented with 10^−6^ mol/l valsartan or PD 98059, a specific extracellular signal-regulated kinase (ERK) inhibitor. The cells were treated for 48 hrs and then used for RT-PCR or Western blotting analysis as described below.

### Histological staining

At the completion of the given observation periods, the rats were killed by intraperitoneally injected with an overdose of 2% sodium pentobarbital, the thoracic aorta tissues were harvested and fixed in 10% formaldehyde for 0.5 hr, then they were embedded in paraffin for preparation of paraffin-embodied sections. Sections were performed with Masson trichrome staining for relative content of smooth muscle and collagen fibre. Morphologic and structural changes of aorta tissue of every group were observed under the standard light microscope in 10 random fields of each section. Photographs were taken by using automatic image processing system (MetaMorph Imaging System; Universal Imaging Corp., Downington, PA, USA).

### Ultrastructure of aorta endothelial cell

The aorta were cut into small pieces and fixed in 2.5% glutaraldehyde in 0.2 M cacodylate buffer (pH 7.4) at 4°C for 2 hrs, then washed in PBS. The materials were incubated in a 2% OsO_4_ solution, dehydrated in a series of increasing ethanol concentrations and propylene oxide, and finally were immersed in Spurr resin. Ultrathin sections (50 nm) were cut on a Leica ultracut UCT ultramicrotome (Leica Microsystems Inc, LKB-II, Wetzlar, Germany), mounted on copper grids, and examined under a JEM 1200EX transmission electron microscope (Jeol, Tokyo, Japan).

### Immunohistochemical staining

Expressions of Bcl-2 and Bax were analysed by immunohistochemical staining. Briefly, the aorta tissue samples were fixed with ice-cold 4% formalin solution in PBS for 10 min. at 4°C. After blocking with 0.2% normal goat serum for 20 min., they were incubated with monoclonal rabbit antibodies against rat Bcl-2 and Bax overnight at 4°C. After extensive washing, they were incubated with a goat antirabbit IgG (Invitrogen, Carlsbad, CA, USA) at room temperature for 1 hr. Bcl-2 and Bax dilutions were used according to the manufacturer guideline.

### RT-PCR

Total RNA from the aorta tissues or cells was extracted with RNAprep pure Cell/Bacteria Kit (TIANGEN, Beijing, China) according to manufacturer's guideline. The purity and yield of RNA were analysed by UV-spectrophotometer (UV 300, Eppendorf, Hamburg, Germany). The value of A260/280 was 1.8–2.0 and 2 μg of total RNAs were used for reverse transcription in a 20 μl reaction with SuperscriptII (reverse transcriptase from Gibco) according to the manufacturer's protocol. 2 μl of the reverse transcripts were used for PCR. The primers used in this study are shown in the Table1.

**Table 1:** Primers used in RT-PCR

Genes	Primers	Size product
AT1R	Sense 5′-CCTACCGCCCTTCAGATAAC-3′	121 bp
	Antisense 5′-TCCTCTGGCTTCTGCTGTCA-3′	
AT2R	Sense 5′-TCTGGCTGTGGCTGACTTACTC-3′	101 bp
	Antisense 5′-CTTTGCACATCACAGGTCCAA-3′	
GAPDH	Sense5′-GCGCCTGGTCACCAGGGCTGCTT-3′	464 bp
	Antisense 5′-TACCGAAGTTGTCATGGATGACCT-3′	
Bcl-2	Sense5′-CCGGGAGATCGTGATGAAGT-3′5	47 bp
	Antisense 5′-ATCCCAGCCTCCGTTATCCT-3′	
Bax	Sense 5′-CCAAGAAGCTGAGCGAGTGTC-3′	377 bp
	Antisense 5′-TGAGGACTCCAGCCACAAAGA-3′	
β-actin	Sense5′-GCCAACCGTGAAAAGATG-3′	701 bp
	Antisense 5′-CCAGGATAGAGCCACCAAT-3′	

Amplification was performed for 35 cycles at 94°C for 40 sec., 63°C for 40 sec. (Bcl-2) or 62°C for 1 min. (Bax and AT1R) or 60°C for 45 sec. (AT2R), 72°C for 1 min. and acquiring at 72°C for 5 min. PCR-amplified DNA was separated on 2% agarose gel, stained with ethidium bromide and visualized and photographed under UV light.

### Western blotting

The aorta tissue or cell samples were homogenized in lysis buffer A (20 mM Tris-HCl, pH8.0, 150 mM NaCl, 1% Triton X-100, 2 mM EDTA, 1 mM phenylmethylsulfonyl fluoride, 20 μg/ml aprotinin, 10 μg/ml leupeptin, 20 mM ß-glycerophate, and 2 mM NaF) for 30 min. The homogenates were centrifugated and protein concentration was determined with BCA protein assay reagent kit (Piece Biotech Inc., Rockford, IL, USA). An equal amount of protein (20 μg/lane for most proteins, while 100 μg/lane for p-p38 and p-JNK detection) from each sample extract was loaded in a 12.5% SDS-PAGE gel for Electrophoresis, and electroblotted onto PDVF membrane. Membrane was blocked with 5% non-fat dried milk (in TBST) for 2 hrs at room temperature and then incubated with primary antibody overnight at 4°C Then, membrane was washed with TBST (10 min. ×3) and incubated with horseradish peroxidase-conjugated secondary antibodies for 1 hr at room temperature (All the antibodies were purchased from Cell Signaling Technology, Boston, MA, USA). After washing with TBST (10 min. ×3), the immunoblots were developed using an ECL Western blotting detection system (Amersham Pharmacia Biotech, Piscataway, NJ, USA) and recorded by exposure of the immunoblots to an X-ray film (Piece Biotech Inc.).

### Statistical analysis

All data were expressed as means ± SD. Differences were evaluated by *t*-test analysis. Statistical significance was defined as *P* < 0.05.

## Results

### Systolic blood pressure, morphological degeneration of aorta and the effect of valsartan

Systolic blood pressure (SBP) of Wistar rats increased with age, from 139.1 ± 6.4 mmHg in the control group to 158.6 ± 5.5 mmHg in the ageing group. In the valsartan group, SBP was 149.0 ± 4.3mmHg (Table2).

**Table 2:** General characteristics of structure and function of the aorta of rats

Rat group	The adult rats (*n* = 20)	The ageing rats (*n* = 20)	The valsartan rats (*n* = 20)
Month (M)	3	18	18
Weight (W)	321.4 ± 31.1	624.9 ± 25.4*	685.7 ± 23.2*
Systolic blood pressure (SBP)	139.1 ± 6.4	158.6 ± 5.5*	149.0 ± 4.3*
T (μm)	53.8 ± 6.2	92.0 ± 15.1*	75.0 ± 10.8*Δ
D (mm)	1.29 ± 0.18	1.65 ± 0.23*	1.51 ± 0.15*Δ
T/D	0.042 ± 0.001	0.056 ± 0.002*	0.050 ± 0.005*Δ
SM (Aa%)	22.5 ± 4.7	32.9 ± 3.4*	23.6 ± 3.0*Δ
CF (Aa%)	21.9 ± 2.6	30.7 ± 3.2*	24.7 ± 2.6**Δ
HC (μg/mg)	12.8 ± 1.4	38.2 ± 15.57*	22.2 ± 6.6**Δ

T: intima-media thickness

D: internal diameter

T/D: intima-media thickness/internal diameter

SM (Aa%): relative content of smooth muscle

CF (Aa%): relative content of collagen fibre

HC: Hydroxyproline.

Mean ± SD, *n*: number of rat per group,

* *P* < 0.05,

** *P* < 0.01 compare with adult group;

Δ *P* < 0.05 compare with ageing group.

To characterize the aorta remodelling, we measured intima-media thickness (T), diameter (D) and T/D, and relative contents of smooth muscle (SM, Aa%) and collagen fibre (CF, Aa%). The results showed that T, D, T/D, SM (Aa%) and CF (Aa%) in the ageing group were significantly higher than that in the control group (*P* < 0.05). However, the above items were all decreased in valsartan treatment group compared with ageing group (data not shown), indicating that valsartan may play a role in preventing the development of aorta ageing.

### Ultrastructural degeneration of aorta with ageing and the effect of valsartan

**Figure 1:**
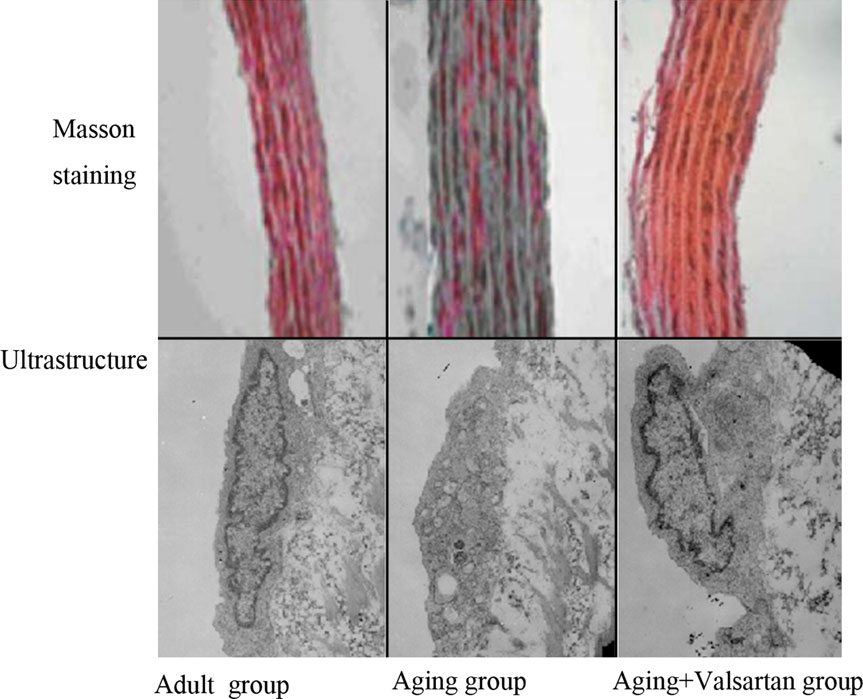
Morphological changes of aorta structure during ageing and beneficial effects of valsartan.

Ultrastructural analysis of aorta was focused on vascular endothelial cells, because the senescent endothelial cells may critically disturb the integrity of the endothelial monolayer and may thereby contribute to vascular injury and atherosclerosis [15–19].The endothelial cell in control group showed a fusiform, smooth shape and even chromatin (Fig.1A). While the endothelial cells in ageing group were flattened and enlarged, there were swollen mitochondria with distorted or lost internal cristae, the volume of the Golgi complex was decreased, and there were abundant unequal vesicles and marrow-like cytolysosome (Fig.1B).

By contrast, valsartan treatment significantly improved the ultrastructure of aorta endothelial cell. In the ageing animals receiving valsartan treatment, a fusiform shape and regularly scattered chromatin appeared in the endothelial cells, where the cytoplasm contained numerous mitochondria, well-developed Golgi complex and abundant ribosome (Fig.1C).

### Expression of Bcl-2 family proteins during ageing and the effect of valsartan

**Figure 2:**
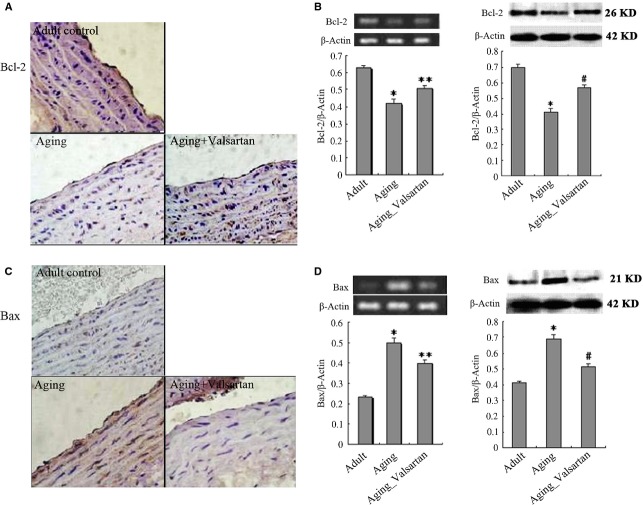
Immunohistochemical staining of Bcl-2 and Bax in aorta endothelium of rats. (A) immunostaining of Bcl-2; (B) Quantitative analysis of Bcl-2 expression by RT-PCR and Western blotting; (C) immunostaining of Bax; (D) Quantitative analysis of Bax expression by RT-PCR and Western blotting. *P < 0.05, **P < 0.01.

The Bcl-2 family has been demonstrated to play an important role in regulation of apoptosis and senescence [20,21]. The ratio of Bcl-2 and Bax has been found to be an important determinant of apoptosis; a high ratio favour cell survival, while a low ratio promotes cell ageing or death [21,22]. Therefore, we determined the expression of Bcl-2 and Bax in aorta in different group. As shown in Figure2, the expressions of Bcl-2 and Bax were examined by immunohistochemical staining, RT-PCR and Western blotting respectively. From immunohistochemical stained sections, we observed that the expression of Bcl-2 was significantly decreased in ageing animals compared with that in adult ones (Fig.2A). Meanwhile, the expression of Bax was significantly increased with ageing (Fig.2C). Quantitative analysis with RT-PCR and Western blotting demonstrated similar results, that expression of Bcl-2 was significantly higher in adult animals than that in ageing ones (*P* < 0.01), while the expression of Bax was significantly lower in adult animals than that in ageing ones (*P* < 0.01). Apparently, the ratio of Bcl-2/Bax in aorta was decreasing with ageing, indicating that ageing resulted detrimental effects on vascular cells. However, the decreased ratio of Bcl/Bax because of ageing could be significantly reversed by long-term administration of valsartan though the value was still significantly lower than that in adult animals (*P* < 0.01). As shown in Figure2, in ageing animals receiving valsartan treatment, the Bcl-2/Bax ratio was significantly improved compared with control ageing animals (Bcl-2 expression increased, *P* < 0.01; while Bax expression decreased, *P* < 0.01). These results suggested that valsartan produced protective effects on aorta cells from ageing.

### AT1R mediates p-ERK activity during ageing

Mitogen-activated protein kinases (MAPKs) are a family of protein-serine/threonine kinases that include at least three distinctly regulated subgroups in mammals: ERK, Jun amino-terminal kinase (JNK), p38MAPK [23]. These enzymes phosphorylate different intracellular proteins and play important roles in regulating cell ageing, survival and death [24,25].

**Figure 3:**
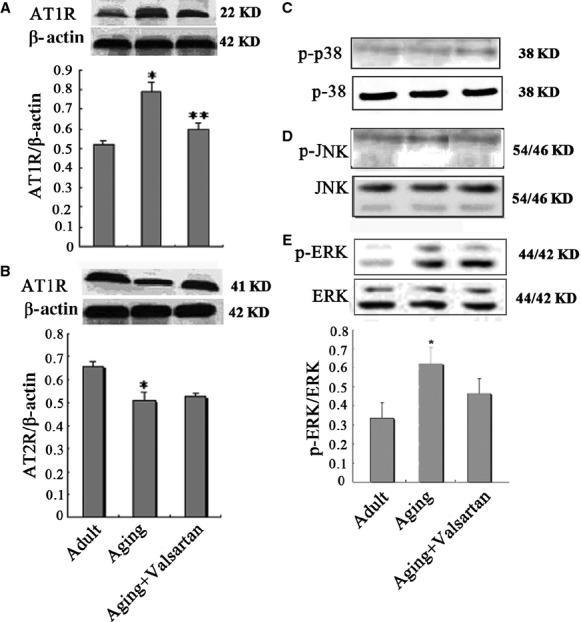
Western blotting analysis of AT1R, AT2R and MAPKs in aorta tissue of rats. (A) AT1R expression in adult, ageing and valsartan groups; (B) AT2R expression in adult, ageing and valsartan groups; (C) Expression of p38, a subgroup of MAPKs in different groups; (D) Expression of p-JNK, a subgroup of MAPKs in different groups; (E) Expression of p-ERK, a subgroup of MAPKs in different groups. *P < 0.01 compared with control group; **P < 0.01 compared with ageing group.

To determine the underlying mechanisms of ageing-induced aorta injury and valsartan exerted protection, we detected the expression of AT1R (the target of valsartan) and the possible involvement of MAPKs during ageing. As shown in Figure3, AT1R expression in ageing animals was significantly higher than that in adult ones (*P* < 0.01), while administration of valsartan significantly reversed ageing-induced increase in AT1R (*P* < 0.01 compared with control ageing animals). However, valsartan produced no effect on the expression of AT2R, indicating the selective blockage of valsartan on AT1R.

**Figure 4:**
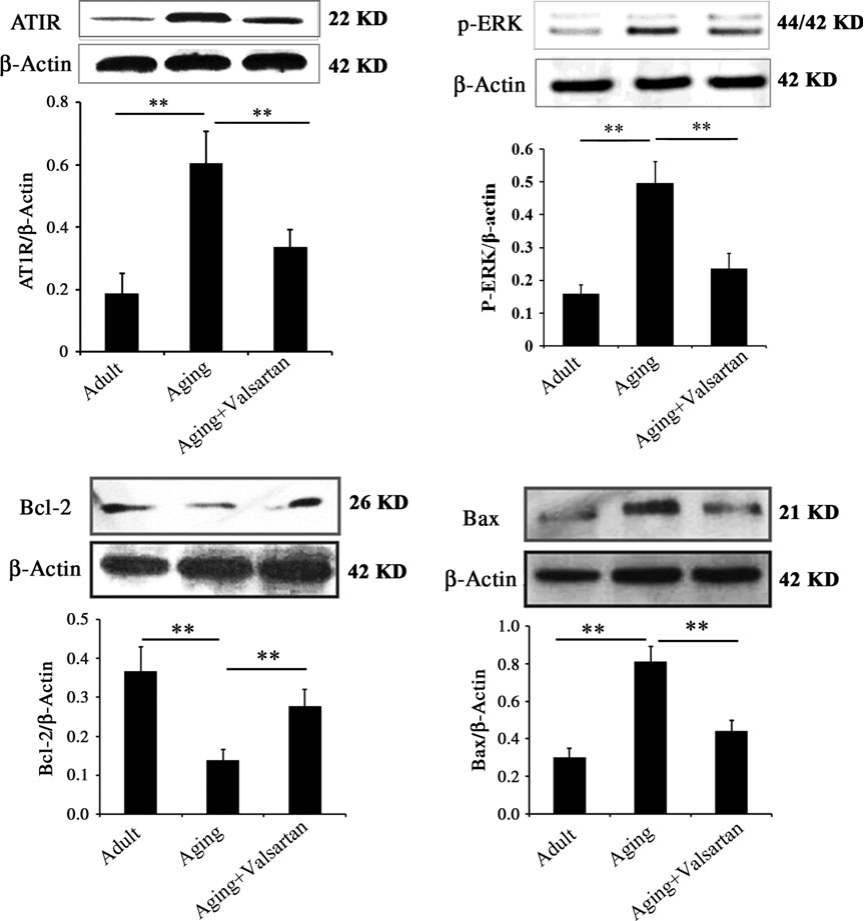
Western blotting analysis of AT1R, p-ERK, Bcl-2 and Bax expression in aorta-derived endothelial cells *in vitro*. Adult indicates endothelial cells derived from adult rats; Ageing indicates endothelial cells derived from ageing rats; Ageing+valsartan indicates valsartan-treated endothelial cells derived from ageing rats. Data are expressed as the means ± SD. *P < 0.01 compared with control group; **P < 0.01 compared with ageing group.

Analysis of three subgroups (ERK, JNK and p38MAPK) of MAPKs demonstrated that both p-JNK and p-p38MAPK were expressed at very low levels in aorta. Compared with adult control, the expression levels of p-p38 and p-JNK seem to be higher in ageing groups, but the valsartan treatment produced no effects on the expression of p-p38 and p-JNK. The results suggested that the protection of valsartan on aorta against ageing is independent of p-p38 and p-JNK (Fig.3C and D). However, ERK activity, that is, p-ERK level significantly increased with ageing (Fig.3E, *P* < 0.01). More importantly, selective blocker of AT1R, valsartan significantly reversed ageing-accompanied increase in p-ERK activity (Fig.3E, *P* < 0.01 compared with control ageing group). These results indicated that AT1R mediated the activity of p-ERK during ageing, which may involve in the ageing-induced aorta injury. To further determine the effects of AT1R on p-ERK, we performed parallel experiments *in vitro*. As shown in Figure4, aorta endothelial cells from adult rats express a low level of AT1R and p-ERK, however, the expression of AT1R and p-ERK were significantly higher in ageing rat-derived aorta endothelial cells (*P* < 0.01). Valsartan treatment significantly decreased the expression of AT1R (*P* < 0.01), accompanying with the decrease in p-ERK (*P* < 0.01). Furthermore, we observed consistent results about the expression of Bcl-2 and Bax with *in vitro* experiment, that is, ageing resulted in the decrease in Bcl-2 and increase in Bax, while valsartan reversed ageing-induced changes in Bcl-2 and Bax (Fig.4C and D). These *in vitro* results provided additional evidence that AT1R mediated the increase in p-ERK activity during ageing.

### AT1R-mediated p-ERK activity involves in the ageing-induced aorta injury

**Figure 5:**
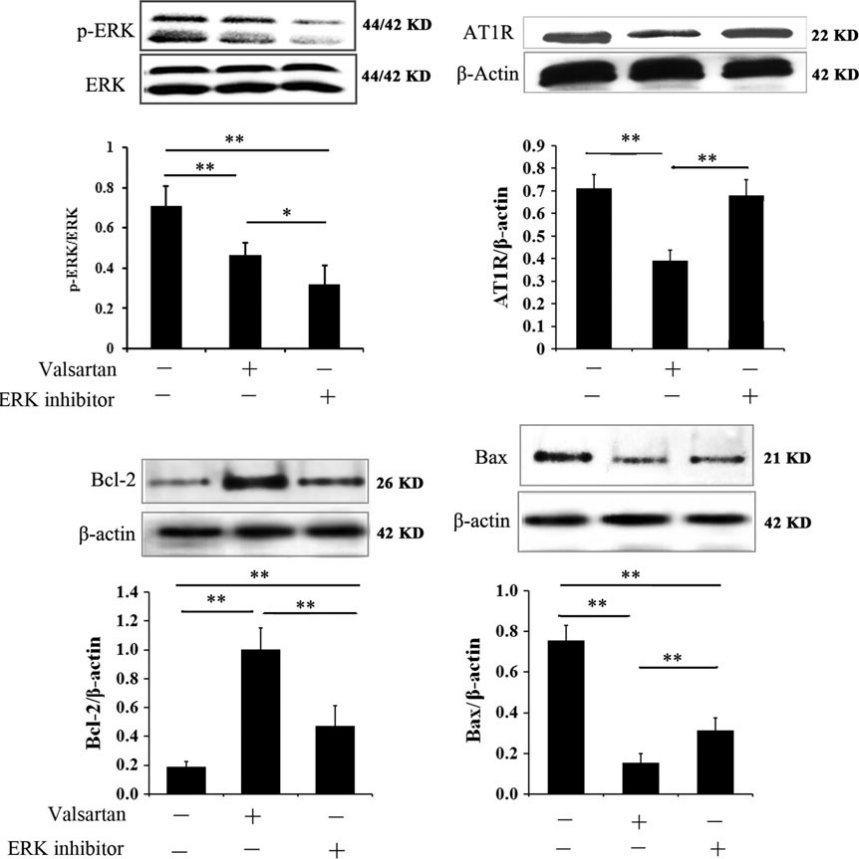
Comparison of valsartan and ERK inhibitor in the protection of ageing aorta cells. The cells used in the experiment were derived from ageing rat aorta, and they were treated with valsartan and ERK inhibitor respectively. Control cells received no treatment. Data are expressed as the means ± SD. *P < 0.01 compared with control group; **P < 0.01 compared with ageing group.

To test whether AT1R-mediated p-ERK activity is correlated with aorta injury during ageing, we investigated the protective effect of ERK inhibitor on ageing rat-derived aorta endothelial cells and compared it with valsartan. As shown in Figure5A, valsartan significantly decreased the expression of AT1R in ageing aorta endothelial cells, while no effect of ERK inhibitor on AT1R was observed. However, both valsartan and ERK inhibitor significantly decreased the expression of p-ERK in ageing aorta endothelial cells compared with control (*P* < 0.01). In terms of Bcl-2 and Bax, we observed that valsartan and ERK inhibitor treatment reversed the expression of Bcl-2 and Bax in ageing aorta endothelial cells in a similar manner (Fig.5C and D). The above results suggested that regulation of p-ERK activity should be an important pathway in the protection of aorta by valsartan during ageing. We could also observe that inhibition of ERK inhibitor on p-ERK activity was more significant than valsartan (Fig.5, *P* < 0.05). However, the protection of ERK inhibitor on ageing aorta was less significant than valsartan. One possible explanation was that AT1R-mediated p-ERK activity may be only one of the mechanisms involving in the pathology of ageing-induced aorta injury, *i.e*. AT1R should also mediate other pathways that contributed to the ageing-induced aorta injury. Therefore, inhibition of AT1R by valsartan was more effective than inhibition of p-ERK by ERK inhibitor in aorta protection.

## Discussion

Cardiovascular disease is the most common cause of death among the elderly, and the ageing is the largest risk factor to the diseases. Accompanying with ageing, lots of adverse changes (or injury) will develop on cardiovascular structure and function, which will directly influence vascular disease threshold and seriousness [2,3,25,26]. Therefore, clarifying the mechanisms underlying the ageing-induced vascular injury is of great significance for prevention and attenuation of such diseases. In this present study, we revealed in the ageing aorta that (*i*) AT1R expression and p-ERK activity in aorta increase with ageing, which may be closely related with structural and functional degeneration of aorta; (*ii*) administration of selective AT1R blocker, valsartan significantly reversed the increase in AT1R occurring with ageing. Simultaneously, p-ERK was also depressed and further, ageing-induced aorta degeneration was improved; (*iii*) ERK inhibitor depressed the level of p-ERK in ageing aorta cells without affecting AT1R, but protective effects on ageing aorta cells were also observed. These data suggested that AT1R-mediated ERK activity was at least one of the important mechanisms in ageing-induced aorta degeneration.

Actually, research on vascular degeneration with ageing is attracting great interest from scientists, but the underlying mechanisms are still not well understood. It has been showed that AT1R mediates most of biological effects, such as vascular constriction, cell proliferation, senescence and reactive oxygen production. Therefore, over the past decades, many studies have focused on the roles of angiotensin system in ageing, especially Ang II. Kosugi *et al*. demonstrated in mouse model that the expression of ageing markers were directly correlated with cardiac Ang II [4]. Yano *et al*. further revealed that up-regulation of AT1R may be involved in the initiation and progression of atherosclerosis [27]. In this study, we demonstrated that AT1R expression in aorta tissue significantly increased, whereas AT2R were significantly decreased compared with that in adult ones (*P* < 0.01). Selective blockage of AT1R significantly reversed aorta degeneration during ageing, indicating the roles of AT1R in the process.

It has been demonstrated that Bcl-2 and Bax genes were critical components for mediating numerous cellular responses, including apoptosis [21,22]. Bcl-2 is an anti-senescence factor, which has been shown to inhibit senescence and apoptosis [28], while Bax was found to promote cell death [29]. Therefore, the ratio of Bcl-2/Bax is closely related to apoptosis. In this study, we found that blocking of Ang II type 1 receptor by valsartan resulted in the significant increase in the ratio of Bcl-2/Bax and decrease in ERK activity in ageing vascular cells, suggesting that both Ang II type 1 receptor and ERK activity may be related to vascular apoptosis during ageing. More interestingly, ERK inhibitor, which did not influence the expression of Ang II type 1 receptor, produced similar effect on the ratio of Bcl-2/Bax as valsartan to some extent. One possible explantation was that Ang II type 1 receptor contributed to the ageing-induced vascular apoptosis and ERK was one of its down effectors, *i.e*. ageing-induced vascular apoptosis partially through AT-1 receptor-mediated ERKs activity.

The activation of MAPKs may vary in different cells under various physiological or pathological conditions [30]. Recent studies have revealed that MAPK signalling transduction pathway is able to mediate senescent signals and regulate ageing process [31,32]. JNK and p38 are important subgroups of MAPKs. In our experiment, we found that it was difficult to detect the expression of p-JNK and p-p38 by Western blotting until much more proteins (about 100 μg) were loaded in electrophoresis. Further, the exposure time of X-ray film was greatly prolonged (about 30 min. or more). We observed that very low levels of p-p38 and p-JNK were detected in vascular cells, this is consistent with the previous reports [33–36]. Compared with adult control, the expression levels of p-p38 and p-JNK seem to be higher in ageing groups. However, the valsartan treatment produced no obvious effects on the expression of p-p38 and p-JNK. The results suggested that the protection of valsartan on aorta against ageing is independent of p-p38 and p-JNK. ERK is another important subgroup of MAPKs, and it has been demonstrated crucial roles in different pathologic processes. In this present study, we observed that the phosphorylation of ERK was associated with ageing and the increase of AT1R. In previous studies, accumulating evidence have identified that the enhanced activity of ERK signalling transduction pathway contributed to the ageing, which was coincident with our research. Further, we observed *in vivo* and *in vitro* that inhibition of AT1R in ageing aorta also inhibited phosphorylation of ERK, while inhibition of ERK produced no effect on AT1R, but both of them produced beneficial effects on ageing aorta, indicating that protection of ageing aorta by AT1R blocker was at the least partially through the activity of ERK.

To further confirm the underlying mechanisms of AT1R-involved aorta degeneration, we further compared the effects of selective AT1R blocker and ERK inhibitor on the protection of aorta during ageing. Though ERK inhibitor was more effective in ERK inhibition than valsartan, it produced significant less effects than valsartan, that is, inhibition of ERK activity could only acquire partial effects as valsartan. A reasonable explantation was that regulation of ERK activity was just one of the mechanisms in AT1R-mediated aorta degeneration during ageing, other mechanisms also existed. These mechanisms are deserved further investigation. However, we could not consider all these mechanisms or mediators in a single study.

In conclusion, we demonstrated that AT1R involved in the ageing-related structural and functional degeneration of aorta, selective blockage of AT1R could significantly attenuate the pathological process, providing a strategy for prevention of ageing-induced vascular diseases and revealing a novel action of valsartan. Further, we demonstrated that AT1R-mediated ERK activity was an important pathway in ageing-induced aorta degeneration, which is of great significance in future developing intervention for preventing or attenuating ageing-related vascular diseases.
